# Factors Affecting Antenatal Care Attendance: Results from Qualitative Studies in Ghana, Kenya and Malawi

**DOI:** 10.1371/journal.pone.0053747

**Published:** 2013-01-15

**Authors:** Christopher Pell, Arantza Meñaca, Florence Were, Nana A. Afrah, Samuel Chatio, Lucinda Manda-Taylor, Mary J. Hamel, Abraham Hodgson, Harry Tagbor, Linda Kalilani, Peter Ouma, Robert Pool

**Affiliations:** 1 Centre for Social Science and Global Health, University of Amsterdam, Amsterdam, The Netherlands; 2 Barcelona Centre for International Health Research (CRESIB), Hospital Clínic-Universitat de Barcelona, Barcelona, Spain; 3 Centre for Global Health Research, Kenya Medical Research Institute, Kisumu, Kenya; 4 Department of Community Health, School of Medical Sciences, Kwame Nkrumah University of Science and Technology, Kumasi, Ghana; 5 Navrongo Health Research Centre, Navrongo, Upper East Region, Ghana; 6 College of Medicine, University of Malawi, Blantyre, Malawi; 7 Department of Infectious & Tropical Diseases, London School of Hygiene & Tropical Medicine, London, United Kingdom; 8 Division of Parasitic Diseases, National Centre for Infectious Diseases, Centers for Disease Control and Prevention, Atlanta, Georgia, United States of America; University of Massachusetts Medical School, United States of America

## Abstract

**Background:**

Antenatal care (ANC) is a key strategy to improve maternal and infant health. However, survey data from sub-Saharan Africa indicate that women often only initiate ANC after the first trimester and do not achieve the recommended number of ANC visits. Drawing on qualitative data, this article comparatively explores the factors that influence ANC attendance across four sub-Saharan African sites in three countries (Ghana, Kenya and Malawi) with varying levels of ANC attendance.

**Methods:**

Data were collected as part of a programme of qualitative research investigating the social and cultural context of malaria in pregnancy. A range of methods was employed interviews, focus groups with diverse respondents and observations in local communities and health facilities.

**Results:**

Across the sites, women attended ANC at least once. However, their descriptions of ANC were often vague. General ideas about pregnancy care – checking the foetus’ position or monitoring its progress – motivated women to attend ANC; as did, especially in Kenya, obtaining the ANC card to avoid reprimands from health workers. Women’s timing of ANC initiation was influenced by reproductive concerns and pregnancy uncertainties, particularly during the first trimester, and how ANC services responded to this uncertainty; age, parity and the associated implications for pregnancy disclosure; interactions with healthcare workers, particularly messages about timing of ANC; and the cost of ANC, including charges levied for ANC procedures – in spite of policies of free ANC – combined with ideas about the compulsory nature of follow-up appointments.

**Conclusion:**

In these socially and culturally diverse sites, the findings suggest that ‘supply’ side factors have an important influence on ANC attendance: the design of ANC and particularly how ANC deals with the needs and concerns of women during the first trimester has implications for timing of initiation.

## Introduction

Antenatal care (ANC), along with family planning, skilled delivery care and emergency obstetric care, is a key element of the package of services aimed at improving maternal and newborn health [1], [2]. In light of evidence from a 2001 systematic review [3], the World Health Organization (WHO) began promoting a new model of ANC for low-income countries, moving away from the traditional model, developed largely in the West. The updated model, based on ‘reduced but goal-orientated clinic visits’ [4], is the so-called ‘focused’ ANC, consisting of (at least) four visits to a health facility during an uncomplicated pregnancy [5]. Although a more recent systematic review has raised questions about the efficacy of focused ANC [6] and revised evidence-based guidelines are being compiled, focused ANC remains the WHO recommendation for low-income countries [4].

The currently recommended focused ANC package incorporates a range of interventions (Table 1) [1]. Clinical research investigating the contribution of components of ANC to improving maternal mortality is ongoing, but some ANC interventions have been shown to be effective for the detection, treatment or prevention of conditions associated with serious morbidity or mortality: monitoring of chronic conditions, anaemia, for example; screening for and treatment of infections, including sexually transmitted infections [7]; prevention of mother-to-child transmission of HIV (PMTCT) [8]; insecticide treated bed nets (ITNs) [9]; and intermittent preventive treatment of malaria (IPTp) with sulfadoxine-pyrimethamine (SP) [10]. Antenatal care is also viewed as an important point of contact between health workers and women and an opportunity for provision of health education – including how to detect pregnancy complications – and development of a birth plan to ensure delivery at a health facility [11].

**Table 1:** WHO-recommended procedures for ANC [1].

Essential	Situational
Confirmation of pregnancy	HIV testing and counselling
Detection of problems complicating pregnancy (e.g., anaemia, hypertensive disorders, bleeding, malpresentations, multiple pregnancy)	Intermittent preventive treatment of malaria (IPT) and promotion of insecticide treated nets (ITN)
Respond to other reported complaints	Deworming
Tetanus immunization, anaemia prevention and control (iron and folic acid supplementation)	Assessment of female genital mutilation (FGM)
Information and counselling on self-care at home, nutrition, safer sex, breastfeeding, family planning, healthy lifestyle	
Birth planning, advice on danger signs and emergency preparedness	
Recording and reporting	
Monitoring of progress of pregnancy and assessment of maternal and foetal well-being	
Syphilis testing	

Although scientific debate concerning the design of ANC continues, research suggests that in low-income countries, particularly sub-Saharan Africa, pregnant women often do not receive the recommended ANC [11], [12]. Across sub-Saharan Africa there is wide variation in ANC attendance: although 71% of pregnant women attend formal ANC at least once, only 44% attend ANC four or more times [12]. To ensure that women achieve four ANC visits and that potential complications are identified in early pregnancy and managed effectively, the WHO recommends that women initiate ANC during the first trimester of pregnancy [13]. However, comprehensive analysis of DHS data from the 1990s suggested that less than 30% of pregnant women achieved this goal [12]. More recent Demographic and Health Survey (DHS) data illustrate that the variation in timing of ANC initiation across sub-Saharan African remains notable: for example, 11% of women started ANC in the first trimester in Ethiopia (2011) [14]; 16% in Nigeria (2008 [15]); 47% in Congo-Brazzaville (2005 [16]) and 55% in Ghana (2008 [17]). Moreover, amongst sub-Saharan countries, the trend over the last ten to 20 years in the proportion of women making at least four ANC visits varies markedly: DHS survey data indicate that in West Africa, eight of ten countries have illustrated increases, whereas, in Southern and East Africa, six of 11 countries have experienced declines [18].

The mismatch between ANC targets and attendance in sub-Saharan Africa has been acknowledged and, in response, social science research aimed at exploring this gap has been conducted. Nonetheless, researchers have tended to rely on questionnaire surveys to collect data on variables that influence ANC attendance [19]. These studies have correlated a range of factors with ANC attendance: for example, a woman’s and/or her husband’s level of education; a woman’s occupation; economic status; distance to health facility; parity; and age [19]. Such statistical associations offer little insight into the mechanism(s) through which such factors operate, neglecting the *how* and the *why*: questions, to which the answers may provide inspiration for possible policy interventions.

A hand-full of studies employing qualitative research methods, such as in-depth interviews, focus group discussions and participant observation, have however directly tackled ANC attendance in sub-Saharan [20]–[23]. For instance, Chapman [20] addressed the question of why pregnant women in a peri-urban settlement of Mozambique with accessible ANC services delayed initiation. This study highlighted the importance of context-specific social factors, such as the, ‘[p]ersonalistic reproductive threats’ that women faced, and illustrates the importance of qualitative research methods and long-term fieldwork to understand behaviour that, without an *insider* perspective, may seem inexplicable. Yet, overall, little qualitative research has directly addressed questions of why women delay ANC in sub-Saharan Africa [20], [24].

In light of the paucity of research (and absence of comparative studies), drawing on the results from a programme of qualitative research, this paper explores the factors affecting ANC attendance in a number of African settings. As a result of its potential impact on the provision of ANC that is in line with WHO recommendations, particular emphasis is placed on investigating ANC initiation, but questions as to why women attend ANC at all are also considered. A comparative approach is taken and data from four research sites (in three countries) are presented; this approach to the analysis of a similar phenomenon, ANC attendance, across diverse social and cultural and healthcare contexts facilitates the identification, and prompts the interrogation, of relevant themes that might otherwise be taken for granted [25]. Furthermore, given that the results of questionnaire studies suggest notable differences in ANC attendance in the three countries (Table 2), this is an opportunity to explore the factors that underpin these differences (as well as differences that occur at each site) and to propose areas for potential policy interventions.

**Table 2:** National and regional data on ANC attendance.

Proportion of women recording:	Kenya (Nyanza Province)^a^	Malawi^b^	Ghana (Ashanti Region; Upper East Region)^c^
**At least 1 ANC visit**	92 (94)	98	92 (92; 97)
**4 or more ANC visits**	47	46	78
**1^st^ visit in 1^st^ trimester**	15	12	55
**1^st^ visit at more than 6 months**	40	38	9

a 2008–2009 [39]; ^b^2010 [36]; ^c^2008 [17].

### Settings

Data were collected in four socially diverse sites across Africa: western Kenya, southern Malawi and northern and central Ghana (Table 3).

**Table 3:** The study settings.

Country	Kenya	Malawi	Ghana	
**Site**	Siaya District(Nyanza Province)	Chikwawa and Blantyre Districts (Southern Region)	Kassena Nankana District (Upper East Region)	Ejisu Juaben and Ahafo Ano South Districts (Ashanti Region)
**Settlements**	Rural (3)	Urban (1)	Urban (3)	Urban (3)
	Peri-urban (2)	Peri-urban (2)	Peri-urban (2)	Peri-urban (2)
		Rural (3)	Rural (2)	Rural (2)
**Health facilities offering ANC**	1 district hospital	1 district hospital	1 district hospital	2 district hospitals
	1 private hospital	1 private hospital	4 main health centres	1 private hospital
	4 small dispensaries/clinics	1 secondary hospital	4 small clinics and CHFP compounds	2 health centre
		6 health centres		4 small clinics
**National ANC user charging policy**	Free	Free	Free	Free
**Focused ANC**	Yes	Yes	Yes	Yes

#### Kenya

Fieldwork was carried out in urban, peri-urban and rural areas of Siaya District, Nyanza Province. Local healthcare facilities and ANC services vary amongst these settlements: urban areas are located within a 30-minute walk to the district hospital, whereas, in rural areas, women mainly access ANC at the small community clinics or dispensaries, which, for some women, are up to two hours’ walk from home. Locally, the principal ethnic group, the Luo, make up over 95% of the population. Livelihood activities include subsistence farming of maize, sorghum, millet and cassava. Due to the relatively limited employment opportunities, migration to urban centres is common, particularly to Kisumu, the nearest city.

#### Malawi

Data were collected in Mpemba and Madziabango, peri-urban areas of Blantyre District, and in rural areas of Chikwawa District, southern Malawi. Three hospitals, and six healthcare centres provide ANC services to the women resident in these areas, who are mainly from the Chewa, Manganja, Sena and Yao ethnic groups. Distances to health facilities providing ANC vary and some women face a three-hour walk (or journey on a bicycle taxi). Women mainly cultivate crops for subsistence and sale at market.

#### Ghana (Ashanti Region)

Although just over half of the population of the Ashanti Region (central Ghana) lives in urban areas (51% in 2000 [26]), both of the districts – Ejisu Juaben and Ahafo Ano South – where data were collected are mainly rural. Furthermore, across the region, agriculture is the main productive activity. Ejisu Juaben District is however more densely populated and closer to Kumasi than Ahafo Ano South. In Ashanti Region, in addition to the majority ethnic group, the Asante, in 2000, 11% of the population were internal migrants [26], mainly from the north and the east. In both districts, data were collected at the district hospitals, two to three health centres, and several smaller clinics. The distances that respondents travelled to health facilities for ANC vary greatly in Ashanti Region. Women often use taxis or minibuses, but those who live furthest from health centres face a walk of up to an hour before being picked up, whereas women who live in the urban centres also use transport to travel the short distance.

#### Ghana (Upper East Region)

Data collection took place in the Kassena-Nankana District, which is located in the Upper East Region, in the north of Ghana. This area borders Burkina Faso and Togo, is part of the Sahel. As there is only one annual rainy season during which people grow millet, maize, and vegetables for subsistence, a large proportion of the population migrate the rest of the year. Upper East is Ghana’s least urbanized region (16% urbanized, in 2000 [26]) and the population is ethnically diverse. However, ethnic groups are concentrated in specific districts: in the Kassena-Nankana District the Kassena and the Nankani make up almost 90% of the population. In Kassena-Nankana District capital (Navrongo) there is a district hospital and outreach community-based services are common through the area. Nonetheless, in Kassena-Nankana, women walk for up to an hour to reach the nearest health facility and longer to a larger health centre.

## Methods

### Ethics Statement

Overall ethics clearance was obtained from the Clinical Research Ethics Committee, Hospital Clinic-University of Barcelona. Separate local ethics clearance was obtained at each site: in Ghana, clearance was obtained from the Institutional Review Board of the Navrongo Health Research Centre, Navrongo and the Committee on Human Research Ethics, Kwame Nkrumah University of Science & Technology, Kumasi; in Kenya, clearance was obtained from the Institution Review Board of Centers for Disease Control and Prevention, Atlanta and from the National Ethics Review Committee, Kenya Medical Research Institute, Nairobi; and in Malawi, clearance was obtained from the College of Medicine Research and Ethics Committee. As approved by all ethics review committees and institutional review boards, informed consent was obtained orally from study participants. Oral rather than written informed consent was obtained because the study procedures posed minimal risk to study participants and to avoid the possible negative influence of a written consent on rapport between researchers and respondents. With the agreement of participants, verbal consent was voice recorded prior to each interview or focus group discussion.

### Data Collection

Data were collected as part of a broader programme of qualitative research investigating the social and cultural context of malaria in pregnancy. In malaria endemic areas, such as the research sites, interventions for the prevention and control of malaria during pregnancy (IPTp and ITNs) are recommended as part of routine ANC at health facilities (see Table 1). As women’s utilization of ANC at health facilities plays a crucial role in uptake of these interventions, exploring factors that influence ANC attendance was therefore a key objective of this programme of research.

The study was coordinated by a team of social scientists, based in Barcelona (Spain), and was conducted in collaboration with local research centres. At each site, fieldworkers, fluent in the local language(s) and with social science research experience, spent extended periods of time in the settlements where data were collected. Members of the Barcelona social science team carried out quarterly field visits to participate in data collection, provide ongoing training, and conduct extended debriefing sessions.

In Kenya, 16 months of fieldwork was carried out between September 2009 and January 2011. In Malawi, fieldwork was divided into two periods: three months (May to August 2009), and nine months (October 2010 to June 2011). In the Ashanti Region, Ghana, fieldwork was conducted between April 2009 and August 2011. Finally in Upper East Region there were two periods of fieldwork: July to December 2009 and April 2010 to March 2011.

Assisted by two Barcelona-based researchers (AM and CP), fieldworkers undertook interviews and focus group discussions in the local language(s) or English depending on the preference of the respondent(s). If the respondent(s) consented, his/her/their responses were recorded and later transcribed verbatim and translated into English for analysis. If a respondent objected to being recorded, detailed notes were taken. Fieldworkers (along with AM and CP) carried out regular field observations in the communities and local healthcare facilities. At each site, a group of pregnant women were selected as case studies and interviewed three to six times pre- and postpartum to elicit their experiences and care seeking as their pregnancy progressed. Individual interviews were commonly conducted in respondents’ homes, or, in the case of health workers, their place of work. Focus group discussions were carried out in community meeting places, usually outside in the shade of a tree.

A flexible and iterative approach was taken to data collection. Starting from broad research questions, the topics explored and questions posed during interviews depended on the themes emerging from the data. Data collection and analysis were carried out in parallel, whereby emerging themes could be identified and incorporated into revised interview guides to provide a comprehensive insight into the relevant topics.

Several techniques were employed to ensure the reliability of the findings: data were triangulated using a range of qualitative methods (interviews, focus groups and observations) with a range of respondents (Table 4). Furthermore, data collection (and analysis) was carried out by more than one member of the research team to reduce the potential for data to be biased by one individual. The case studies also provided a useful opportunity to develop rapport with respondents and confirm the accuracy of responses from previous interviews.

**Table 4:** Number and type of respondent by study site.

Method	Respondent type	Ghana (Ashanti Region)	Ghana (Upper East)	Kenya	Malawi	Total
Case studies	Pregnant women	19	18	12	18	67
In-depth individual interviews	Pregnant women	84	64	69	68	285
	Health providers*	33	34	17	21	105
	Relatives	26	29	20	16	91
	Opinion leaders	12	12	10	12	46
Focus group discussions	Community members	10	16	9	16	51

* Includes health care staff involved with the provision of ANC at health facilities and TBAs working in the communities.

### Respondents

Across the sites, to ensure that a range of perspectives were incorporated and hence improve the reliability of findings, group and individual interviews were conducted with pregnant women and other types of respondent. A range of strategies was therefore employed to identify potential respondents.

In the first instance, pregnant women resident in the research sites were identified either with the assistance of community leaders (that often had contacts with the collaborating local research centres) or at the health facility when attending ANC. In addition, snowball sampling was utilized: pregnant women introduced the researchers to pregnant friends or relatives. Using purposive sampling, efforts were made to ensure that research staff interviewed both married and unmarried pregnant women of a range of ages, parities and gestational ages from across the different settlements (within the field sites). Women who were identified in early pregnancy were enrolled as case studies. Ultimately, the number of pregnant women interviewed was a result of the directed sampling and the point of saturation, whereby no further novel insights were identified from interviews.

A point of saturation approach was also applied to the total sample of healthcare workers, local opinion leaders and the relatives of pregnant women. Health workers involved in ANC were identified at the local health facilities, whereas other healthcare providers, such as traditional birth attendants (TBAs), were identified through contacts with other respondents. Opinion leaders (community leaders and elders) were also identified through snowball sampling and contacts with local leaders.

### Data Analysis

Data from each site were first analyzed separately and then combined. Using Atlas Ti 6 (Scientific Software, Berlin, Germany), codebooks were developed, in collaboration with the fieldworkers/social scientists, using a combination of established categories based on the original research questions (examples of basic broad codes included ‘first ANC’, ‘IPTp’ etc) and codes that emerged from the data using a Grounded Theory approach [27]. The codebooks were flexible and codes were reassessed during data collection and revised according to the emergence of novel themes. Based on discussion between CP and AM, data from the four sites at key codes relevant to ANC attendance (e.g. ‘pregnancy disclosure’, ‘first pregnancy’) were extracted and collated. Data from the four sites at these different codes were then compared and situated in the site-specific data.

## Results

The findings presented are based on individual in-depth interviews with 285 pregnant women, 105 health providers (involved with the provision of ANC at health facilities or TBAs), 91 relatives and 46 opinion leaders. A further 51 focus group discussions with community members were carried out and 67 women were recruited as case studies and interviewed at least three times (see Table 4 for site-specific details). The findings are also informed by analysis of the observations carried out at each site and recorded as field notes by members of the research team. Throughout the following section, for reasons of brevity, the sites are referred to as ‘Kenya’, ‘Malawi’, ‘Ashanti Region’ and ‘Upper East Region’. This shorthand should not however be interpreted as any attempt at regional or national generalization.

### Women’s Perceptions of ANC and Reasons for Attending

Although women’s descriptions of ANC varied across and within the sites, on the whole, they did not recall receiving all WHO-recommended procedures (Table 1). The descriptions were also often vague and focused on the *experience* of procedures, such as receiving injections or tablets, rather than their aim or purpose. Kenyan women focused on palpation, receiving ‘blood booster’ tablets and injections and were generally less familiar with other procedures or their purpose (such as IPTp). Ghanaian and Malawian women emphasized being weighed (in Malawi and Upper East Region, Ghana ANC was termed, ‘scale’), and also commonly recalled checking the position of the baby, and the provision of medicines and injections. In Malawi, women distinguished ‘blood pills’ from malaria drugs, and recalled being given ITNs. Women in Ghana reported having their arms ‘tied’, but did not explicitly link this with blood pressure measurement. Women described being injected and tested, but specific mentions of HIV testing were only made frequently in Malawi, and references to syphilis tests and haemoglobin analysis were rare overall. Indeed, interviews with health workers and observations indicated that, often as a result of shortages or infrastructure problems, not all the recommended ANC procedures were carried out for every woman or at every healthcare facility. Lack of delivery of specific procedures, such as syphilis testing and haemoglobin analysis, therefore influenced women’s descriptions of ANC.

At all the sites, women stated that they attended ANC to monitor the progress of their pregnancy or to check the position of the unborn child. In Upper East Region, women attended ANC to identify problems during pregnancy, whereas, in the Ashanti Region, women also highlighted the importance of taking the medicines provided during ANC to ensure the health of the pregnancy and the development of the baby. Furthermore, Ghanaian respondents, particularly in the Ashanti Region, viewed ANC as a normal part of pregnancy: attending the clinic was simply what women did. In Upper East Region, ANC was often considered compulsory: a result of the authority of health staff or the vague idea of it being the ‘law’. Also linked to the authority of health staff, in Kenya, obtaining an ANC (or ‘birth’) card motivated attendance. The cards, completed by health staff, contain details of ANC attendance and Kenyan respondents suggested that without the cards, they would encounter problems if they attended a health facility to deliver: women feared being reprimanded by healthcare staff, or refused care. Although this played a lesser role in Ghana and Malawi, reference was also made to ANC cards’ importance for avoiding conflicts with health staff.

### Gestational Age and the Timing of ANC Initiation

Both health staff and other community members confirmed that for women at all sites, gestational age was a meaningful concept and influenced ANC attendance. Although their estimations were not always accurate, women talked about the gestational age of their pregnancies – often measuring the progression in months – and reported that this affected when they initiated ANC. Although primagravidae, particularly young women and adolescents, were less certain (as is elaborated below), generally, women became aware of their pregnancy as a result of one or two months of amenorrhea. However, gestational age had a varied impact on ANC initiation across the sites: respondents from the different categories tended to characterize women in Ghana as generally starting ANC around the third or fourth month of pregnancy, whereas women in Kenya and Malawi were often reported to make their first visit at around the sixth or seventh month.

### Reproductive Concerns and Uncertainties

Previous or ongoing health problems – pregnancy-related or otherwise – prompted women to seek care at a health facility in early pregnancy (the first or early second trimester). In Ghana, generally, women initiated ANC in early pregnancy and, from the first visit, ANC was conducted in a problem-focused manner: health workers reportedly paid attention to women’s complaints and possible remedies. Malawian and Kenyan women who complained of ill health during early pregnancy would however generally not attend ANC but rather seek care at a health facility, without disclosing their pregnancy to staff. Yet, at all the sites, experiences of previous pregnancy complications motivated women to seek ANC in early pregnancy.

Although women described how a couple of months of amenorrhea was generally sufficient to confirm a pregnancy, both health staff and pregnant women reported that, at health facilities, palpation was often used to confirm pregnancy at 12 weeks. Pregnancy tests were available in the larger health facilities at all the sites, but they were often prohibitively expensive, particularly in Kenya and Malawi (around $2 in Kenya). Therefore, generally pregnancies were not confirmed with a test, except in district hospitals in Ghana, where pregnancy tests were used in cases of uncertainty. This uncertainty in the first trimester, prior to palpation, extended to both the woman and the health staff. In Malawi and Kenya, this had implications for ANC attendance: as is explored below, there were reports of health workers instructing women to return when they were able to confirm a pregnancy (or the pregnancy was confirmed elsewhere) and perform ANC procedures.

Any uncertainty around pregnancy status was pronounced for women who had previously had difficulties conceiving or bringing a pregnancy to term. Given the central role that reproduction often plays in the women’s lives and the stigma that surrounds infertility, including the implications that childlessness have for a woman’s relations with a woman’s husband and in-laws, for these women, confirming a pregnancy was particularly important. In Malawi, and to a lesser extent in Ghana, there was also uncertainty about pregnancy linked to the use of traditional and modern methods of contraception. In these three sites, confusion about amenorrhea associated with injectable contraceptives resulted in women being unclear about their pregnancy status and in some instances led to delayed ANC. In Ghana, health professionals linked irregular menstruation and uncertainties regarding pregnancy to sexually transmitted infections. The uncertainty and ambiguity surrounding pregnancy, particularly in the first trimester also had implications for pregnancy disclosure, as detailed below.

### Parity, Age and Pregnancy Disclosure

Parity had a complex impact upon ANC initiation. For example, unaccustomed to the experience of pregnancy, the associated signs and symptoms, some primagravidae were more likely to seek advice and assistance and initiate ANC earlier. However, this lack of familiarity with the signs of pregnancy also prompted uncertainty: less likely to recognize a pregnancy, they were more prone to unintentionally delay ANC. Nonetheless, these decisions were not taken alone and on the basis of advice from older women, primagravidae hastened their first ANC visit. For example, if a mother became aware of her daughter’s pregnancy – and, on occasion, this seemingly occurred before the adolescent realized herself – she would assist her in attending ANC as soon as possible.

For primagravidae, pregnancy disclosure influenced timing of ANC. Across all the sites, all types of respondent reported that adolescents and unmarried younger women hid their pregnancies and delayed ANC to avoid the potential social implications of pregnancy: exclusion from school, expulsion from their natal home, partner abandonment, stigmatization and gossip. In contrast, older women did not make active efforts to hide their pregnancies. However, they would only directly disclose their pregnancy to close relatives and their husband. Although ambivalent towards others discovering their pregnancy, which they considered inevitable as the pregnancy progressed, women were wary to be accused of boastfulness by spreading the news openly.

Limited pregnancy disclosure was generally reported as a means to avoid gossip and potential embarrassment if a woman did not bring her pregnancy to term. In Malawi, however, there were reports of women delaying pregnancy disclosure and ANC (till the fourth month) to avoid suffering witchcraft that could harm a pregnancy. In Kenya and in Ghana, pregnant women (and other community members) described how they were at greater risk of witchcraft and sometimes attributed pregnancy interruptions to witchcraft. However, this was not viewed as a reason to delay ANC. Furthermore, in Ghana, although women acknowledged the dangers of witchcraft and personalistic threats to a pregnancy (threats posed by human or non-human sentient beings), they were reticent to talk about them.

At all the sites, disclosure was a particularly sensitive issue for women who had experienced unexplained pregnancy interruption. For example, although one Kenyan woman, who had previously experienced several unexplained pregnancy interruptions, was willing to be interviewed in early pregnancy, she had not informed her closest friends and neighbours. Later, she reported having lost the pregnancy, and although she did not refuse outright to be interviewed, henceforth, whenever approached, she did not have time to talk.

In spite of the concerns about gossip, embarrassment and witchcraft, it was possible to identify and interview women during early pregnancy. Contact was made at health facilities, or with the assistance of community leaders or other pregnant women. Although the numbers varied across the sites – from five in Kenya to twelve in Malawi – in total, over 30 women were interviewed during the first trimester of pregnancy.

With regard to older multiparous women, health workers could confirm that particularly in Kenya and Malawi, and to a lesser extent in Ghana, they visited the clinic in later pregnancy: in some instances, waiting till the ninth month. Being more accustomed to the pregnancy experience, their priority was obtaining the antenatal card and they were less concerned about monitoring the progress of the pregnancy.

### Interactions with Healthcare Workers

Pregnant women’s interactions with healthcare staff at ANC had varying implications for ANC attendance. Respondents (including pregnant women, their relatives, community members and opinion leaders) reported that delaying ANC until the third trimester, led to chastisements from health workers; this was particularly the case if a woman arrived at a health facility to deliver without having previously attended ANC. Hence, as previously described, women’s fear of chastisement from health workers sometimes prompted ANC attendance.

Women’s interactions with healthcare staff could also result in delayed ANC. The most extreme examples in Kenya involved one direct and one indirect report of women who attended ANC in the first trimester, but were sent home and instructed to return in the second trimester, when their pregnancy was visible and could be confirmed through palpation. These reports from pregnant women conflicted with the statements of Kenyan healthcare staff who said that they encouraged pregnant women to attend ANC as soon as they realize that they are pregnant. In Malawi, during data collection, three women were referred to the hospital from a health centre because the health staff were unable to confirm a pregnancy. Furthermore, during health talks Malawian health staff did not advise women on when to initiate ANC, but when such messages were given, generally, women were advised to initiate ANC in their third month and only rarely did a staff member state that women should start as soon as they realize they are pregnant. In both sites in Ghana, women were generally advised to attend ANC as soon as they realized that they were pregnant and none of the observed women that attended a health facility for ANC during the first trimester were sent back home.

In spite of the messages and reprimands that women experienced, healthcare workers’ advice was generally trusted and women claimed to follow their instructions. This is epitomized by women’s attitude to follow-up ANC appointments: the scheduling was viewed as compulsory. Furthermore, observations confirmed that communication between women attending ANC and the health staff was limited and often didactic. In Kenya and Malawi, health education was provided in groups and although during the ANC visits there were opportunities for dialogue with healthcare staff, observations suggested that pregnant women rarely took advantage of this. In Ghana, however, health talks were given less often and information was provided on a one-to-one basis during consultations. Moreover, during these consultations, health staff asked women directly about their health concerns. Healthcare staff explained that, as a result of the transition to focused ANC, information was no longer provided to pregnant women during health talks.

Interactions between pregnant women and health workers during ANC were also influenced by social factors. At health facilities, communication tended to be more two-way if a woman was comparatively wealthy or well educated or had a familial relationship or friendship with the health worker. Members of the research team observed such women addressing health staff on relatively equal terms. This contrasted with the typically quiet, reserved, *head-down* demeanour of other women when interacting with health staff during ANC. Kenyan women also reported chastisements, and social discrimination at health facilities if their birth spacing was deemed inadequate. Some women with young children would therefore avoid attending health facilities, and this could lead to delaying their first visit. In contrast, although at the other sites, appropriate birth spacing was described as important, women did not mention having a young child as a reason to delay ANC.

### The Direct and Indirect Costs of ANC

In Kenya, from observations and interviews with pregnant women, it was apparent that charges for ANC varied across health facilities and amongst respondents: small charges were levied for the ANC card and also, where available, laboratory tests. Similarly, and in spite of the free health insurance for pregnant women, in the Ashanti Region, incidences of charging for some ANC services were reported: although, not encountered in all facilities, the pricing system was unclear and consequently the subject of women’s complaints. Furthermore, health staff described the efforts of local health administrations to tackle corruption and prosecute those responsible. In contrast, in Upper East Region, ANC was largely free. However, in some instances, as a result of shortages, women were required to bring with them medical supplies, such as bottles for sampling urine. In Ghana, ITNs were offered at a subsidized charge of 2$ for pregnant women and there were regular shortages. Although charges were not levied for ANC visits in Malawi, women were instructed to buy replacement generic health passports due to a shortage of ANC cards.

Attending ANC also entailed indirect costs. Travel costs varied amongst the sites and the respondents at each site: for example, in northern Ghana, where vehicles providing public transport were scarce, women mainly walked to the clinic and travel costs were minimal. In Kenya and Malawi, bicycle taxis were available, and in light of their pregnancy-related tiredness, women who could afford to pay, did so. Other women travelled on their husband’s bicycle and, in Kenya, a minority of women used motorbike taxis because of their greater comfort. Other indirect costs include the food that women purchased whilst waiting to be attended, either for themselves or their accompanying children. Given the particularly social nature of ANC visits, women with the available resources spent money on clothes and a visit to the hairdresser prior to attending (all women however made efforts to look smart). Many of the women cultivated land along with their husbands and other family members and were often responsible for cooking meals for family members; taking time out from these activities therefore represented an opportunity cost. There are also non-monetary costs: pregnancy, combined with women’s continued labour demands (that continue up to delivery and recommenced shortly after), was often an exhausting experience for women and the journey to health facilities represented a physical burden.

Delays in ANC initiation were not however solely due to the associated indirect and direct costs. The nature of ANC appointment scheduling by health staff, and women’s understanding of appointments as compulsory also contributed to delayed initiation, particularly in Kenya. In Kenya and Ghana, both women and health staff described how follow-up appointments were generally scheduled for one month after each appointment, except in the weeks prior to their due date, when women were scheduled for weekly or fortnightly visits. In Malawi, appointments were every two months except during the ninth month. Women, particularly in Kenya and Malawi, reported that they would not attend ANC until the sixth or seventh month to minimize the number of journeys and therefore the total cost of ANC. As women viewed the scheduled appointments as compulsory, attending in the third month of pregnancy could potentially result in eight journeys to the health facility (assuming that in the final month a fortnightly appointment is set and excluding delivery at a health facility).

A range of factors also mediated women’s access to the means necessary to meet the direct and indirect costs of ANC. At all the sites, women were primarily involved in subsistence farming, but, through their involvement in small businesses, some were able to gain access to cash. Women without direct access to cash often relied on their husbands or relatives to meet costs, which further complicates decision-making about ANC initiation. In some instances, however, it was not only a question of access to cash, but also access to the means of transport, such as a husband’s bicycle, to reach the health facility. Reports of women delaying ANC initiation because of an objection from their husbands or a relative responsible for household expenditure were however rare. The difficulties that some women face to access cash were highlighted by the experience of one Kenyan woman who worked as a live-in carer: she reported waiting for her employer, who knew of her pregnancy, to pay her salary before initiating ANC.

### Husbands and HIV-related Stigma

In Malawi and Kenya, health staff promoted the involvement of husbands in ANC through, for example, giving preferential treatment and a free shawl for their child if the husband attended ANC with his wife. For a minority of Kenyan women, however, the participation of husbands in ANC decision-making, combined with HIV-related stigma, had negative implications for their ANC attendance: women were wary of attending ANC because they would be informed of their HIV status and a positive result had ramifications if their husbands discovered their status. Husbands often refused to be tested and rather, in the most extreme instances, accused their wives of adultery and abandoned them. In light of this, one of the Kenyan case studies reported delaying ANC to delay discovering her HIV status. This was possible, because although HIV/AIDS was not mentioned on the ANC card, people knew how to interpret the information available on the card to determine HIV status and one Kenyan woman had attempted to damage her ANC card to hide her status. Furthermore, Kenyan women were reticent to talk about HIV testing: unless specifically asked, they would not mention it as part of ANC. Although there were also reports of HIV-related stigma in Malawi, in general, Malawian women described the importance of knowing their status and HIV testing was not given as a reason to delay ANC. In Ghana, the HIV prevalence in the research sites was much lower and HIV/AIDS was not raised as an issue affecting ANC attendance.

## Discussion

National and regional data on ANC attendance illustrate varying trends across sub-Saharan Africa [12]. Generally, women attend ANC at least once, as findings of this study confirm. Nonetheless, across the research sites, survey data indicate two notably different patterns of ANC attendance: on the one hand, over half of Ghanaian women attend ANC in the first trimester of pregnancy and less than 10% initiate ANC in the third trimester; whereas, in Kenya and Malawi, 12% and 15% of women, respectively, initiated ANC in the first trimester and around 40% in the third trimester (Table 2). Data collected for this study also resonated with these patterns of ANC attendance. Most strikingly, these patterns were each observed in two socially and culturally distinct sites: two very different Ghanaian sites, on one hand, and a site in Malawi and Kenya, on the other. However, variations in ANC attendance were also observed amongst women at each site (age and parity were identified as key factors that influenced timing of ANC initiation) suggesting that women’s timing of first ANC visit is subject to complex influences.

### What Ideas do Women have about ANC and why do they Attend at All?

Overall, in spite of some variation within and between the sites, women only had a vague understanding of specific ANC procedures. This finding resembles the results of other qualitative research conducted in Ashanti Region, Ghana: women viewed ANC as a package and the details of specific procedures were ancillary [28]. This vagueness is not surprising given the inconsistent delivery of ANC procedures (due to a lack of facilities or women’s inability to meet charges): a finding also highlighted in Burkina Faso, Uganda and Tanzania [29], [30]. A potential contributing factor is the limited information that women received during health talks and one-to-one consultations, which has also been reported in other parts of sub-Saharan Africa, where ANC was described as a ‘missed opportunity’ to inform women about possible pregnancy complications [31]–[34].

This limited understanding influenced women’s motivations for attending ANC: they had general ideas about caring for their pregnancy, such as checking the foetus’ position or monitoring its progress, that echo findings from other qualitative research [21]. Additional social pressures were also reported: particularly, in Kenya (as in Tanzania [23]), avoiding reprimands from health staff when they attended a health facility for delivery – through obtaining an ANC card – was important; the insults that women feared had social implications (embarrassment and shame) but refusal of care was also a concern. For Ghanaian women, ANC was normalized in the Ashanti Region or viewed as compulsory in Upper East Region.

### Explaining the Pattern of ANC Attendance Across the Sites

Compared to attending ANC at least once during pregnancy, a more complicated array of factors influenced the timing of ANC initiation: reproductive concerns and uncertainties; parity, age and pregnancy disclosure; interactions with health staff; and the direct and indirect costs of ANC. Explaining how these factors contribute to the observed pattern of ANC initiation across the four sites is therefore challenging. Nonetheless, the findings suggest that Ghanaian women were more likely to attend ANC in the first trimester as a result of a combination of the following inter-related reasons.

In Ghana, ANC services were better orientated towards dealing with early pregnancy and the uncertainty surrounding pregnancy: pregnancy tests were more accessible – albeit only at the district hospitals – and therefore women and health staff, in cases of uncertainty, were able to confirm a pregnancy and did not have to wait to palpate. Faced with a confirmed pregnancy, health workers were therefore able to begin offering ANC. However, even if a pregnancy was not confirmed, ANC in Ghana was continued because emphasis was placed on dealing with women’s health problems instead of, as the cases of women who were turned away from ANC in Kenya and Malawi suggest, a focus on carrying out the ANC procedures on a confirmed, visible pregnancy. Although in Ghana communication problems were observed, this problem-focused strategy was facilitated by the one-to-one nature of communication between pregnant woman and health workers that had replaced the health talks. Moreover, in light of this problem-focused design and the ability to confirm a pregnancy in the first trimester, health staff were more able to provide clear messages about attending ANC in the first trimester and less likely to deter women from attending prior to palpation. This meant that illness during early pregnancy prompted women to access ANC in Ghana, whereas women in Malawi and Kenya, who also usually sought care for illness during early pregnancy, tended to do so at a health facility without disclosing their pregnancy and this has potentially important implications for the delivery of pharmaceuticals that are contraindicated during pregnancy. Additionally, in both sites in Ghana there were particularly strong social pressures to initiate ANC in early pregnancy, which was either viewed as compulsory or normalized amongst women.

In spite of the overall earlier initiation of ANC among Ghanaian women, although outside the immediate scope of this study, it is notable that, both nationally (and particularly in Upper East) relatively few women deliver at a health facility. Indeed, national survey data from low-income countries illustrate the relationship between ANC attendance and place of delivery [35] and this complexity is highlighted by the contrast between Ghana and Malawi: Ghanaian women initiate ANC earlier and are more likely to achieve four ANC visits, yet the proportion of deliveries in health facilities is lower: 58% of deliveries in Ghana [17] and 75% in Malawi [36]. This suggests the need for further analysis of women’s transition from pregnancy to delivery care.

### Explaining Variations in ANC Attendance at Each Site

At all sites, age and parity had complex impacts on ANC. Primagravidae’s lack of experience of pregnancy either hastened initiation – seeking assistance, in some cases, as a result of advice from relatives – or delayed initiation – not realizing that they were pregnant or not disclosing their pregnancy. Indeed, at all sites, some adolescents and unmarried younger women hid their pregnancies and delayed ANC to avoid the social ramifications of pregnancy at their age, mainly expulsion from school. Given the specific social repercussions of pregnancy at this age and the fact that this analysis did not focus specifically on this age group, further exploration of the factors influencing ANC attendance and pregnancy care amongst adolescents and young women is also needed. Older multiparous women, particularly in Kenya and Malawi, visited the clinic towards the end of the second trimester; as has been reported in South Africa [37], older respondents, more accustomed to the pregnancy experience, prioritized obtaining the ANC card and were less concerned about receiving assistance in monitoring their pregnancy. However, multiparous women who had experience previous health problems during pregnancy were likely to initiate ANC earlier.

Although, the data suggest that older women did not actively hide a pregnancy, disclosure was limited to their husbands and close relatives. These reports of limited disclosure are similar to those described elsewhere in Mozambique [20] and Zimbabwe [21]: ambivalent towards others noticing their pregnancy, which was considered inevitable, women were wary of seeming boastful as a result of spreading the (good) news openly. Furthermore, in The Gambia, particularly among young women, such concerns about gossip led women to hide their pregnancy and delay ANC, as this was seen as a clear sign of pregnancy [38]. However, in part, the findings contrast with those of Chapman [20] and Mathole et al. [21], who described how, in light of their reproductive vulnerability in early pregnancy linked to personalistic threats and witchcraft, women made active efforts to hide their pregnancies and, hence, delayed ANC. Although women did not actively hide their pregnancies or delay ANC due to the risk of witchcraft at all sites, women were said to be particularly vulnerable to such threats, and limited pregnancy disclosure was linked explicitly to concerns about witchcraft in Malawi. In Ghana, women were particularly reticent to talk about such threats and, generally, women reported limited disclosure to avoid the embarrassment that they would experience if they did not bring the pregnancy term.

Women’s (and their husbands’) socio-economic status and level of education have often been associated with higher levels of formal ANC [19]. Although this study did not approach such associations in a quantitative manner, the data suggest insights into the relationship between wealth and/or education and ANC. Women who are relatively wealthy or able to access familial wealth are less likely to be perturbed by the greater total cost of ANC associated with initiation in early pregnancy. The data also suggest that a woman’s level of education plays an important *social* role; secondary or tertiary education enables women to approach health staff on relatively equal terms, to pose questions and, potentially, to seek care with lesser concern about any possible reprimands. Given the greater social capital that comparatively wealthy and educated women could call upon in the resource-poor research sites, they were also less likely to fear the social ramifications normally associated with health workers’ chastisements. Particularly as these women are often friends, acquaintances or relatives of health staff and if necessary can utilize private healthcare facilities, where they would not tolerate admonishments.

### Policy Considerations

Given the cultural diversity across the sites, the findings suggest a prominent role for the influence of *supply* side factors on ANC initiation. Altering the *design* of ANC could therefore promote earlier initiation. Such modifications should however take into account local ideas about pregnancy care and the contexts in which their decisions take place. Reproductive concerns and uncertainties are manifested differently – across contexts, between age groups and parities and amongst individuals – and incorporating these concerns and uncertainties is key to developing appropriate ANC. Nonetheless, from this comparative analysis, several key areas identified as affecting ANC initiation could be targeted for interventions.

### Uncertainty During the First Trimester

The capacity of ANC service design to meet the needs of women in the first trimester influences timing of ANC initiation. Women often face uncertainty and vulnerability in the first trimester, especially adolescents, young women and primagravidae, and women who have previously suffered reproductive interruptions. Therefore, improving the accessibility of pregnancy tests has the potential to reduce this uncertainty amongst women (e.g. linked to the use of injectable contraceptive) and health staff.

### Women’s Health Problems, Particularly during the First Trimester

In addition to the uncertainty of early pregnancy, during this time women reported health problems but did not necessarily start ANC, instead they sought assistance at a health facility without disclosing a suspected pregnancy. Providing ANC that focuses on pregnant women’s health concerns, and allowing women to communicate their concerns to health staff, particularly during the first trimester, may encourage women to start ANC earlier.

### Women’s Interactions with Health Staff

Health staff who provide ANC exercise significant authority and women generally place trust in the instructions they give. Therefore, messages about when to attend ANC communicated by health staff seemingly influence ANC attendance and ambiguous instructions can result in delays to ANC. Women may not disclose early pregnancy to non-ANC health staff and this has potential implications for the delivery of contraindicated medications.

### Other Actors that Influence ANC Attendance

Given the influence of friends and relatives on decision-making regarding ANC, in terms of offering advice or supplying resources to meet the overall costs of care, messages about ANC attendance would be more effective if aimed at the community as a whole.

### Inflexible Monthly Follow-up Appointments and Focused ANC

From many women’s perspective, a lack of flexibility with regard to monthly scheduled follow-up appointments increases the total number of visits and therefore the total cost of ANC, which has particular impacts for women with limited resources and large distances to travel to health facilities. To ensure that women attend ANC in early pregnancy a balance has to be struck between ensuring that women return for follow-up appointments during their pregnancy and an awareness of the maximum number of journeys to the health facility that they are able to afford. Furthermore, this inflexibility illustrates that WHO recommendations for *focused* ANC were interpreted in different ways: often, rather than focusing on a minimum of four visits, health staff continued to follow the previous version of ANC, with monthly visits scheduled.

### Direct Charges of ANC

Although not authorized in national ANC policy, charges are levied for ANC procedures. These charges add to a range of other costs that women have to meet when attending ANC and lead to delays in attendance and reduced compliance with the WHO recommended ANC procedures. Ensuring the local implementation of national ANC user charging policies is therefore recommended.

### The Stigma of Adolescent Pregnancy

In light of the social ramifications of pregnancy at this age, most prominently expulsion from school, adolescents and young women are at particular risk of delaying pregnancy disclosure and ANC. Developing strategies that enable pregnant adolescents to access ANC without experiencing stigma could promote earlier ANC initiation. Efforts to mitigate the social ramifications of adolescent pregnancy, such as exclusion from the education system, may also prompt earlier pregnancy disclosure and ANC.

### Complacency among Older Multiparous Women

Older multiparous women were at particular risk of delaying ANC. Developing ANC to meet their needs and care preferences, combined with messages about the dangers of complacency, may also promote earlier ANC amongst this group.

### Strengths and limitations

The use of qualitative methods, in combination with long-term data collection, enabled analysis of *how* a range of factors influences ANC attendance, rather than simply providing associations between social and/or economic variables and ANC attendance. Moreover, the analysis of data from several sites – with a combination of varied social and cultural contexts, and varied and similar ANC delivery and attendance profiles – meant that the interaction of factors associated with the delivery of, and the demand for, ANC could be explored. Indeed, the comparative approach taken, ensured that neither *supply* nor *demand* side factors were taken for granted, but rather interrogated and analyzed in combination. Hence the social and cultural contexts of ANC delivery and uptake were explored together and compared and contrasted across the sites.

The findings are limited by the fact that exploring ANC attendance was a supplemental objective of the programme of research, which was primarily orientated towards investigating the social and cultural context of malaria during pregnancy. For example, during observations of ANC, emphasis was placed on assessing the delivery of interventions for the prevention and control of malaria in pregnancy rather than other interventions. Nonetheless, in spite of this, sufficient data on ANC were collected to enable a thorough comparative analysis of the factors influencing attendance.

### Conclusion

This paper has explored factors affecting ANC attendance across four settings that demonstrate two distinct patterns of ANC attendance. In these socially and culturally diverse sites, the findings suggest that both demand *and* supply side factors have an important influence on ANC attendance. Timely ANC attendance was influenced by: women’s and health staff’s uncertainties in early pregnancy; the design of ANC and its capacity to deal with uncertainty around pregnancy status and the degree to which care is orientated towards women’s health concerns; the provision of clear, unambiguous recommendations about the timing of ANC and messages that identify ANC as a service that deals with health concerns during early pregnancy; and the perceived normality of ANC initiation in early pregnancy. Furthermore, a perceived lack of flexibility regarding follow-up appointments increased the total cost of ANC, which can result in delayed ANC, particularly, amongst women with limited resources and who face high transport costs. Moreover, the direct charges levied for ANC procedures – not authorized in national ANC policy – represented only part of the wider cost of ANC. Adolescents and young women were at particular risk of delaying ANC initiation and further research should focus on this group. To ensure appropriate design and effective delivery of ANC, attention should be paid to the *on-the-ground* implementation of ANC and women’s understanding of these local forms of ANC at health facilities, how women deal with reproductive uncertainty and the efforts that women make to care for themselves and their pregnancies.
